# The effect of a school-based intervention on physical activity, cardiorespiratory fitness and muscle strength: the School in Motion cluster randomized trial

**DOI:** 10.1186/s12966-020-01060-0

**Published:** 2020-11-26

**Authors:** Elin Kolle, Runar Barstad Solberg, Reidar Säfvenbom, Sindre M. Dyrstad, Sveinung Berntsen, Geir K. Resaland, Ulf Ekelund, Sigmund A. Anderssen, Jostein Steene-Johannessen, May Grydeland

**Affiliations:** 1 Department of Sports Medicine, Norwegian School of Sport Sciences, Ullevål Stadion, PB 4014, 0806 Oslo, Norway; 2 Department of Physical Education, Norwegian School of Sport Sciences, Ullevål Stadion, PB 4014, 0806 Oslo, Norway; 3 Faculty of Health Science, Department of Public Health, University of Stavanger, Forus, PB 8600, 4036 Stavanger, Norway; 4 Faculty of Health and Sport Science, Department of Sport Science and Physical Education, University of Agder, PB 422, 4604 Kristiansand, Norway; 5 Center for Physically Active Learning, Faculty of Education, Arts and Sports, Western Norway University of Applied Sciences, Campus Sogndal 6856 Bergen, Norway; 6 Department of Physical Performance, Norwegian School of Sport Sciences, Ullevål Stadion, PB 4014, 0806 Oslo, Norway

**Keywords:** Physical activity, Fitness, Adolescents, Randomized controlled trial

## Abstract

**Background:**

Physical activity (PA) declines throughout adolescence, therefore PA promotion during this period is important. We analyzed the effect of two school-based PA interventions on daily PA levels, cardiorespiratory fitness (CRF) and muscle strength among adolescents.

**Methods:**

For the nine-month School in Motion intervention study (ScIM), we cluster-randomized 30 Norwegian secondary schools (*N* = 2084, mean age [SD] = 14 [0.3] years) to one of three study arms. The physically active learning (PAL) intervention included 30 min physically active learning, 30 min PA and a 60 min physical education (PE) lesson per week. The Don’t worry-Be happy (DWBH) intervention included a 60 min PA lesson and a 60 min PE lesson per week, both tailored to promote friendships and wellbeing. Both intervention arms were designed to engage the adolescents in 120 min of PA per week in addition to recess and mandatory PE lessons. The control group continued as per usual, including the standard amount of mandatory PE. PA (main outcome) was assessed by accelerometers, CRF and muscle strength (secondary outcomes) were assessed by an intermittent running test and selected tests from the Eurofit test battery.

**Results:**

Daily PA and time spent in moderate- to vigorous-intensity PA (MVPA) decreased in all groups throughout the intervention. The mean difference in PA level and MVPA for participants in the PAL-intervention arm was 34.7 cpm (95% CI: 4.1, 65.3) and 4.7 min/day (95% CI: 0.6, 8.8) higher, respectively, compared to the control arm. There were no significant intervention effects on daily PA level, MVPA or time spent sedentary for adolescents in the DWBH-intervention arm. Adolescents in the PAL-intervention arm increased distance covered in the running test compared to controls (19.8 m, 95% CI: 10.4, 29.1), whilst a negative intervention effect was observed among adolescents in the DWBH-intervention arm (− 11.6 m, 95% CI: − 22.0, − 1.1).

**Conclusion:**

The PAL-intervention resulted in a significantly smaller decrease in daily PA level, time spent in MVPA, and increased CRF compared to controls. Our results indicate that a teacher-led intervention, including three unique intervention components, is effective in curbing the decline in PA observed across our cohort and improving CRF.

**Trial registration:**

ClinicalTrials.gov ID nr: NCT03817047. Registered 01/25/2019 ‘retrospectively registered’.

**Supplementary Information:**

The online version contains supplementary material available at 10.1186/s12966-020-01060-0.

## Introduction

Throughout adolescence, physical activity (PA) levels are known to decline corresponding with an increase in time spent sedentary [1, 2]. Accelerometer-assessed PA data from a national cohort in Norway showed that 50% of 15-year-olds met the recommended level of 60 min of moderate- to vigorous-intensity PA (MVPA) per day [3]. At the same time, the adolescents spent approximately 70% of their awake time sedentary, and were thereby more sedentary than the retired population [4]. This is of concern as participation in regular MVPA during adolescence is associated with improved physiological and psychological health [5]. Strong inverse associations have also been reported between cardiorespiratory fitness (CRF) and clustering of cardiovascular risk factors in children and youth [6], and lower physical fitness is detrimentally associated with obesity in childhood [7]. Further, positive associations have been reported between both PA, CRF and academic performance [8, 9]. PA levels during childhood have also been found to predict PA levels in adulthood, which support the idea that enhancement of PA in children and adolescents is of importance for the promotion of public health [10, 11]. Hence, there is a need to develop and evaluate interventions focusing on PA and fitness in the young population.

Comprehensive school-based PA interventions have been endorsed by both health and education authorities as a strategy for promoting PA [12, 13], yet the effects these interventions have on young people’s PA and fitness levels are uncertain. In a recently published meta-analysis, it was concluded that current school-based efforts do not positively impact young people’s accelerometer assessed daily PA level [14]. Most school-based PA interventions are implemented among children in primary school. Fewer PA interventions have been carried out among adolescents in lower secondary schools, and the generalizability of these studies are limited, for instance only examining girls [15, 16] or boys [17], or being implemented among adolescents in a middle-income country [18] or low-income or disadvantaged communities [15, 17, 19]. Further, the intervention components vary markedly making comparisons between studies difficult.

Due to the limited evidence but great potential of school-based PA interventions, we conducted a cluster randomized trial titled School in Motion (ScIM), which included two PA intervention arms. Both interventions aimed at increasing PA among adolescents in lower secondary schools receiving two additional hours of PA per week compared to a non-intervention control arm. The primary aim of this paper was to assess the effectiveness of these interventions on adolescents’ daily mean PA levels (main outcome). The secondary aims were to assess the effect of the interventions on PA levels during school hours, CRF and muscle strength.

## Methods

### Study design

ScIM was a multicenter, school-based, three-arm cluster randomized controlled trial (RCT) recruiting ninth graders from lower secondary schools. The study was conducted by four collaborating study partners with a geographical spread across Norway (Norwegian School of Sport Sciences, Western Norway University of Applied Sciences, University of Agder, and University of Stavanger). A random sample of lower secondary schools located in municipalities near the four study partners were included. We excluded private schools, designated special schools, schools with less than 25 adolescents in ninth grade, and schools that worked systematically with PA as an integrated part of the school day.

Schools were randomized manually by a lottery to one of the following three study arms: the intervention arm “Physically Active Learning” (PAL) (*n* = 10), the intervention arm “Don’t worry-Be happy” (DWBH) (*n* = 10) or the control arm (*n* = 10) in a 1:1:1 ratio. The randomization was stratified by district (study center) to ensure that schools from all four study locations were represented in each of the three study arms. The data-manager who conducted the randomization did not participate in any other aspects of the study. The randomization process took place after inclusion but prior to baseline testing. Neither participants, schools nor researchers were blinded.

The project was reviewed by the Regional Committee for Medical and Health Research Ethics (REK) in Norway, who according to the Act on medical and health research (the Health Research Act 2008) concluded that the study did not require full review by REK. The study was approved by the Norwegian Centre for Research Data. The design, conduct, and reporting of this trial adhere to the CONSORT statement. The CONSORT checklist can be found in Additional file 1 [20], and the TIDieR checklist can be found in Additional file 2.

### Interventions

The ScIM intervention arms were designed to engage the adolescents in 120 min of PA per week in addition to their mandatory physical education (PE) lessons (approximately 120–180 min per week) and recess periods. Schools in the two intervention arms added 60 min of PA and 60 min of PE to the class schedule per week. This was achieved by redistributing 5 % of lesson time to PA from other subjects in the curriculum (corresponding to 60 min of PA per week), while the other 60 min were added to the weekly lesson schedule. Thus, for students in the intervention schools, the school week increased by 60 min. All intervention schools received financial resources from The Norwegian Directorate for Education and Training to account for increased expenses. The amount received was based on the number of students attending the school (approximately $90 per student). For all ninth-grade students attending an intervention school, participation in the intervention components was mandatory. The interventions were delivered from September 2017 to June 2018.

The PAL-intervention included three components: 1) An additional lesson of PE per week (60 min), including activities according to the curriculum, planned and taught by a PE teacher. The PE teacher used these lessons to grade the students; 2) a 30 min/week lesson of physically active learning where play-based activities were integrated into other curriculum subjects (i.e. math, English, Norwegian). The aim was to increase PA levels among students while improving their academic performance. The classroom teacher for the subject planned and taught the lesson; and 3) a 30 min/week lesson of PA that included a variety of activities, preferably of at least moderate intensity, and it should be enjoyable. A classroom teacher or a PE teacher planned and taught this lesson. In the PAL-intervention, the 60 min PE lessons included time for teachers to organize activities and for the adolescents to change clothes and shower.

The DWBH-intervention arm included two components; a “Be happy” lesson and a “Don’t worry” lesson (each lesson was 60 min/week). At the start of the intervention, the adolescents formed groups of 3–8 students based on their hobbies. The groups could comprise students from different school classes. Examples of activities chosen were traditional sports (e.g., football or handball), lifestyle sports (e.g., parkour or BMX cycling), dancing, and outdoor recreation. The groups were expected to perform the chosen activity in the “Be happy” lesson throughout the intervention period. In the “Don’t worry” lesson, the students returned to their normal classes and either continued or introduced their class peers to their “Be happy” activity. If the students had been doing group activities in the “Don’t worry” lesson, such as handball or football, they instead practiced relevant skills in the “Don’t worry” lesson. For example, throw technique, endurance and/or strength training for a handball player. Consequently, for the “Don’t worry” lesson the gym would be full of students performing different activities. The “Don’t worry” lesson was conducted as an ordinary PE lesson such that the standard PE curriculum was applied, and the PE teacher used these lessons to grade the students. “Be happy” lessons were mandatory though not graded. In DWBH, both intervention components were planned and led by the students while the teacher(s) was (were) present for support if needed. Depending on the chosen activity, both intervention components included time for the students to organize the activities, change clothes and shower.

### Theory

When planning and developing the interventions, we applied the socio-ecological framework modelled by McLeroy [21]. In order to change behavior, we can approach the individual at different levels: some structures are close to the individual (e.g., individual preferences and social relationships with family and friends), and some structures are more distal, such as community infrastructure and legislation. These factors are all potential domains for effecting behavior change, and positive changes in facilitators on all levels will in theory promote actual behavior change. In the interventions we targeted all levels, though we could influence some levels more than others: individual; (promote motivation for PA through mastery and enjoyment of the activities); interpersonal (promote PA among friends); organizational (promotion of PA through the school as a structure, however we made no physical changes of the school yard to promote PA due to lack of budget); community level (no specific intervention); public policy (adding extra PE in the government set school curriculum). Hence, changes at all levels can promote positive and lasting change in health behavior. In terms of individual and social factors, the PAL-intervention builds on Harter’s competence motivation theory [22], Bandura’s social-cognitive theory [23] and Ryan & Deci’s self-determination theory [24]. The rationale is thought to function as a mediating structure between intervention strategies and outcomes.

The DWBH-intervention arm was anchored to an integrative relational developmental systems (RDS) approach to human development [25], theories on Positive Youth Development (PYD) [26] and the concept of Positive Movement Experiences (PME) [27]. By letting the adolescents choose their own activity, the intention was for them to engage in an activity that was meaningful for them with friends. According to the theories on PYD all youths have strengths. Therefore, in the DWBH-intervention arm the adolescents were responsible for conducting the intervention. They had to form activity groups, determine group aims for the activities, develop a management structure, a strategy for impending conflicts and routines to register attendance.

The schools in the control arm continued current practice including the usual amount of mandatory PE that was part of the curriculum.

### Delivery

Each week teachers in the intervention schools documented the extent to which the intervention dose was delivered as intended using an online form. They reported if the intervention component was delivered, as well as the intensity and the duration of the activity. The intensity was judged subjectively by the teacher who delivered the component. The information from this subjective assessment of intensity is on a group level and is therefore limited as it cannot be used when analyzing the results. However, it provided an indication of the general intensity of the activities.

### Assessment and measures

All participants were tested using an identical set of outcome measures at baseline and follow-up. Baseline measurements (both accelerometers and physical fitness tests) were done in April to June 2017 while students were in eighth grade. The follow-up measures were taken approximately 12 months after the baseline measures, while the participants were in the last phase of the intervention. A team of researchers visited each school in the study and collected all data while the participants were at school. All research personnel were thoroughly trained prior to the data collection by members of the research team.

### Physical activity level (primary outcome)

PA was assessed by triaxial accelerometry (ActiGraph GT3X+, LLC, Pensacola, Florida, USA). The adolescents were instructed to wear the accelerometer on the right hip during all waking hours (except during swimming/bathing) for seven consecutive days. Accelerometers were initialized to start recording at 6.00 AM on the day after they were distributed. The epoch length was set to 10 s. We used the ActiLife software to initialize and download the accelerometer files (version 6.13, ActiGraph, LLC, Pensacola, Florida, USA). All raw accelerometer files were processed and analyzed using specifically developed and commercially available software (StataCorp. 2015. Stata Statistical Software: Release 15. College Station, TX: StataCorp LP). A variable for “school hours” was created by matching the timetable provided by each individual school with the accelerometer file. A valid day was defined as wear time of ≥480 min/day accumulated between 06:00 and 24:00, whereas a valid school day was defined as wear time of ≥40% of school hours. Non-wear time was defined as at least 20 consecutive minutes of zero counts [28]. Participants with at least 2 of 7 valid days and at least 2 of 5 valid school days of activity recordings were included in the analysis on daily PA and school day, respectively. The outcome variables were daily mean activity counts per minute (cpm), as well as time spent sedentary and in MVPA. We defined sedentary time as all activity below 100 cpm, and MVPA as all activity > 2000 cpm. The latter cut-point was developed for the European Youth Heart Study and is equivalent to a walking speed of adolescents of > 4 km/h [2]. Time spent in MVPA or being sedentary was determined by summing total minutes, where the count met the criterion for that intensity divided by the number of valid days of recording, giving an average (min/day) across the assessment period.

### Cardiorespiratory fitness (secondary outcome)

We used the Andersen test to assess the participants’ CRF [29]. The Andersen test is an intermittent running field test lasting for 10 min. The reproducibility of the Andersen test is considered good (r = 0.84), and the association between running distance in the Andersen test and VO_2_max measured on the treadmill have shown a correlation coefficient of 0.60 among 14-year-old elite football players [29]. We administered the Andersen test as per standard procedures indoors on a wooden or rubber floor, however, due to different sizes of available indoor facilities we standardized the length to 16 m (original protocol 20 m). All adolescents were tested in groups of 6–12 individuals. The test required the participants to run back and forth between two lines 16 m apart for a total of 10 min, with 15 s work periods and 15 s breaks standing still. Each time the adolescents turned around at the end line, they had to touch the floor with one hand. The aim of the test was to cover the longest possible distance during the 10-min run, and the participants were meant to run to voluntary exhaustion. Adult test assistants, who subjectively judged whether the child completed a valid test (judging whether the students worked hard enough), recorded the distance covered. The distance covered (in meters) was used as a proxy for cardiorespiratory fitness.

### Muscle strength (secondary outcome)

Muscle strength (i.e., endurance, isometric and explosive strength) was measured using reliable and validated tests selected from the Eurofit test battery [30]: 1) Upper limb strength – handgrip strength was measured using a handheld dynamometer (Baseline® Hydraulic Hand Dynamometer, Elmsford, NY, USA) (measured in kg, best of result of two attempts was used); 2) Explosive strength in the lower body was measured using a standing broad jump test (measured in cm, the best result of two attempts was used); and 3) Abdominal muscle endurance was tested using a sit-up test (number of correctly performed sit-ups within 30 s).

### Height and weight

The adolescents wore light clothing with footwear removed. Weight was measured to the nearest 0.1 kg with a digital scale (Seca 889, SECA GmbH, Hamburg, Germany). We subtracted 0.6 kg (light clothing) or 1.5 kg (more heavy clothing) from each person’s weight to account for clothing. Height was measured to the nearest 0.1 cm using a portable stadiometer (Seca 213, SECA GmbH, Hamburg, Germany) with the individual standing upright facing forward. Body mass index (BMI) was calculated as weight (kg) divided by the height squared (m^2^).

### Socioeconomic status

We used the highest education level of each participant’s parents (whichever was the higher) as a proxy for socioeconomic status (SES). Data regarding parent’s education level came from registry data collected by Statistics Norway. Four SES groups were computed: low (primary school, lower secondary school, vocational high school), middle (secondary school/high school), middle high (undergraduate degree) and high (graduate degree).

### Sample size calculation

Power calculations were conducted to determine the required sample size for detecting changes in the primary outcome (daily mean PA level [cpm]). The ScIM study was designed to detect a difference in mean PA level of 7 % (49 cpm) between the participants in the intervention arms and the control arm. We assumed a standard deviation (SD) of 150 cpm, a power of 90%, a significance level of 0.05, which lead to 492 individuals in each group. To allow for 20% loss to follow-up we needed 590 individuals in each group. We required a minimum of ten clusters per study arm and consequently we aimed to recruit clusters and individuals until we had at least ten clusters and 590 individuals in each arm.

### Statistical analyses

Participants with valid data on PA, CRF or muscle strength at either baseline or follow-up were included in the analysis. To examine whether missing data were missing at random, or completely at random, we conducted the missing at random including covariates test in Stata (mcartest) with main outcome (cpm) and intervention model, with gender, height and weight as covariates (in order to test for covariate-dependent missing). The test supports the missing at random assumption (*p* = 0.096), although the evidence against missing at random is not very strong. Analyses were assessed for assumptions of normality and homogeneity of variance. Descriptive data are presented as means and SDs. Baseline differences between participants in the three study arms were investigated using linear regressions adjusted for gender. We fitted linear mixed models to all continuous outcomes with repeated measurements. Each model contained fixed effects for intervention, time (baseline – follow-up) and intervention x time interaction. As the units of randomization were schools, we added random effects for school, class and subject ID to accommodate the clustering of participants within these units. Based on the linear mixed models, we estimated mean group values with 95% CI at baseline and follow-up. We estimated the between group difference in change from baseline to follow-up between the participants in the intervention arms and the control arm, with adjustment for gender. A value of *p* < 0.05 was considered statistically significant. We examined whether gender modified the intervention effect by introducing an interaction term (timepoint x group x gender). Statistically significant interactions between genders were evident in all PA, CRF and muscle strength models (*p* ≤ 0.003 for all interactions), consequently we repeated the analyses stratified by gender. Data were analyzed using Stata (StataCorp. 2015. Stata Statistical Software: Release 15.1. College Station, TX: StataCorp LP).

## Results

**Fig. 1:**
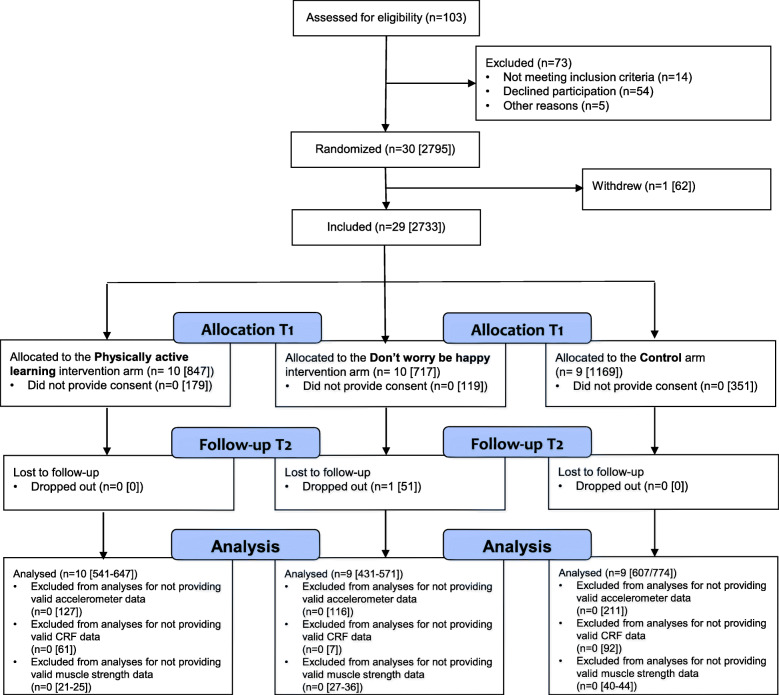
Flow of schools and participants through the study. All numbers are schools [participants]. T1 = baseline; T2 = follow-up

Thirty out of 103 invited lower secondary schools agreed to participate (Fig. 1). One of the included schools in the control arm withdrew from the study after the randomization procedure but prior to baseline testing, leaving nine schools in the control arm. A total of 2084 adolescents (intervention arms: *n* = 1266), which represented 76% of those eligible, provided parental consent, and completed baseline testing. Three months into the intervention period, one school in the DWBH-intervention arm withdrew from the study due to practical reasons. A total of 1579 of the included participants had valid PA assessments at baseline and/or follow-up and were included in the analyses for the primary outcome. For the secondary outcomes a total of 1873 participants had valid CRF assessments and 1976–1992 participants had valid muscle strength assessments at baseline and/or follow-up (handgrip: *n* = 1992; standing broad jump: *n* = 1976; sit-ups: *n* = 1977). Participants included in the analyses were comparable to those excluded in terms of all variables of interest (data not shown).

Baseline characteristics of the participants who were assessed are outlined in Table 1. Students in the PAL-intervention arm were on average 1.1 cm shorter (*p* = 0.005), and students in the DWBH-intervention arm were on average 1.8 kg heavier (*p* = 0.003) compared to students in the control arm. Table 2 shows the participants’ PA levels at baseline. Both during the full day and during school hours students in the PAL-intervention had a significantly lower mean PA level, spent more time sedentary and less time in MVPA than students in the control arm (*p* ≤ 0.002). Further, students in both intervention arms had significantly lower CRF and completed fewer sit-ups within 30 s at baseline than their peers in the control arm (*p* ≤ 0.001) (Table 3). For the standing broad jump test, students in control group jumped 4 cm longer than the students in the PAL-intervention (*p* = 0.003).

**Table 1:** Participant characteristics at baseline by study arm (*N* = 1981). Results are presented as mean (SD) unless otherwise stated

	PAL-group	DWBH-group	Control
N	647	577	757
Age (years)	13.9 (0.3)	14.0 (0.3)	14.0 (0.3)
Sex (% girls/boys)	50/50	49/51	49/50
Height (cm)	164.6 (8.1)	166.4 (7.7)	165.8 (7.7)
Weight (kg)	54.2 (10.8)	56.2 (11.0)	54.4 (10.5)
BMI (kg/m^2^)	19.9 (3.1)	20.2 (3.2)	19.7 (3.1)
**Socioeconomic status**^**a**^			
Primary school (%)	6.5	6.5	5.0
Upper Secondary school (%)	27.1	31.5	28.3
University < 4 years (%)	42.8	39.4	41.6
University > 4 years (%)	23.6	22.5	25.1

*PAL* Physically active learning; *DWBH* Don’t Worry – Be Happy, *BMI* Body mass index

^a^Based on parental education

**Table 2:** Mean (95% confidence interval) physical activity level among participants stratified by study arm at baseline and follow-up

	PAL-intervention		DWBH-intervention		Control	
	Baseline	Follow-up	Baseline	Follow-up	Baseline	Follow-up
**Physical activity** **levels full day**						
N	500	317	406	229	566	288
Wear time (min/day)	776 (765, 787)	763 (751, 776)	775 (763, 787)	768 (754, 782)	770 (759, 781)	742 (729, 754)
Average PA (cpm)	510 (479, 541)	497 (464, 531)	531 (497, 565)	476 (439, 513)	537 (505, 569)	489 (455, 524)
MVPA (min/day)	67 (63, 72)	65 (60, 70)	70 (65, 75)	63 (57, 68)	71 (66, 76)	65 (60, 70)
Sedentary time (min/day)	541 (533, 548)	553 (545, 561)	534 (526, 542)	559 (550, 567)	531 (523, 538)	548 (540, 557)
**Physical activity** **school hours**						
N	536	375	427	284	583	353
Wear time (min/day)	322 (313332)	312 (303, 322)	321 (311, 331)	323 (312, 333)	314 (304, 324)	292 (282, 302)
Average PA (cpm)	444 (398, 489)	462 (415, 508)	504 (456, 553)	411 (361, 461)	501 (455, 547)	433 (385, 481)
MVPA (min/day)	25 (21, 28)	25 (22, 28)	28 (25, 32)	24 (20, 28)	29 (25, 32)	24 (20, 27)
Sedentary time (min/day)	227 (221, 232)	230 (228, 240)	217 (211, 223)	234 (228, 240)	220 (215, 225)	227 (222, 233)

*PAL* Physically active learning; *DWBH* Don’t Worry – Be Happy, *cpm* Counts per minute; *MVPA* Moderate- to vigorous-intensity physical activity. All analyses are adjusted for gender, wear time (except cpm), school cluster, class cluster and subject ID as random effect

**Table 3:** Mean (95% confidence interval) for cardiorespiratory fitness and muscle strength among participants stratified by study arm at baseline and follow-up.

	PAL-intervention		DWBH-intervention	Control		
	Baseline	Follow-up	Baseline	Follow-up	Baseline	Follow-up
Cardiorespiratory fitness (m)	894 (869, 919)	925 (900, 950)	909 (883, 934)	909 (883, 935)	928 (903, 954)	940 (915, 966)
Handgrip (kg)	30 (28, 32)	32 (30, 34)	30 (28, 30)	32 (30, 34)	29 (27, 31)	32 (30, 34)
Standing broad jump (cm)	168 (166, 171)	176 (173, 179)	171 (169, 174)	178 (175, 181)	172 (170, 175)	179 (176, 181)
Sit, ups (n)	18.3 (17.4, 19.2)	19.6 (18.7, 20.5)	18.3 (17.4, 19.2)	18.9 (218.0, 19.9)	19.4 (18.5, 20.3)	20.1 (19.2, 21.1)

*PAL* Physically active learning; *DWBH* Don’t Worry – Be Happy. All analyses are adjusted for gender, school cluster, class cluster and subject ID as random effect

During the intervention period, 83 and 78% of the intervention dose was delivered in the PAL-arm and the DWBH-arm, respectively. This means that the schools in the PAL-intervention arm delivered an average of 100 min/week of additional PA lessons, whereas the schools in the DWBH-intervention arm delivered an average of 94 min/week of additional PA lessons.

### Primary outcome - physical activity level

#### Daily PA

**Fig. 2:**
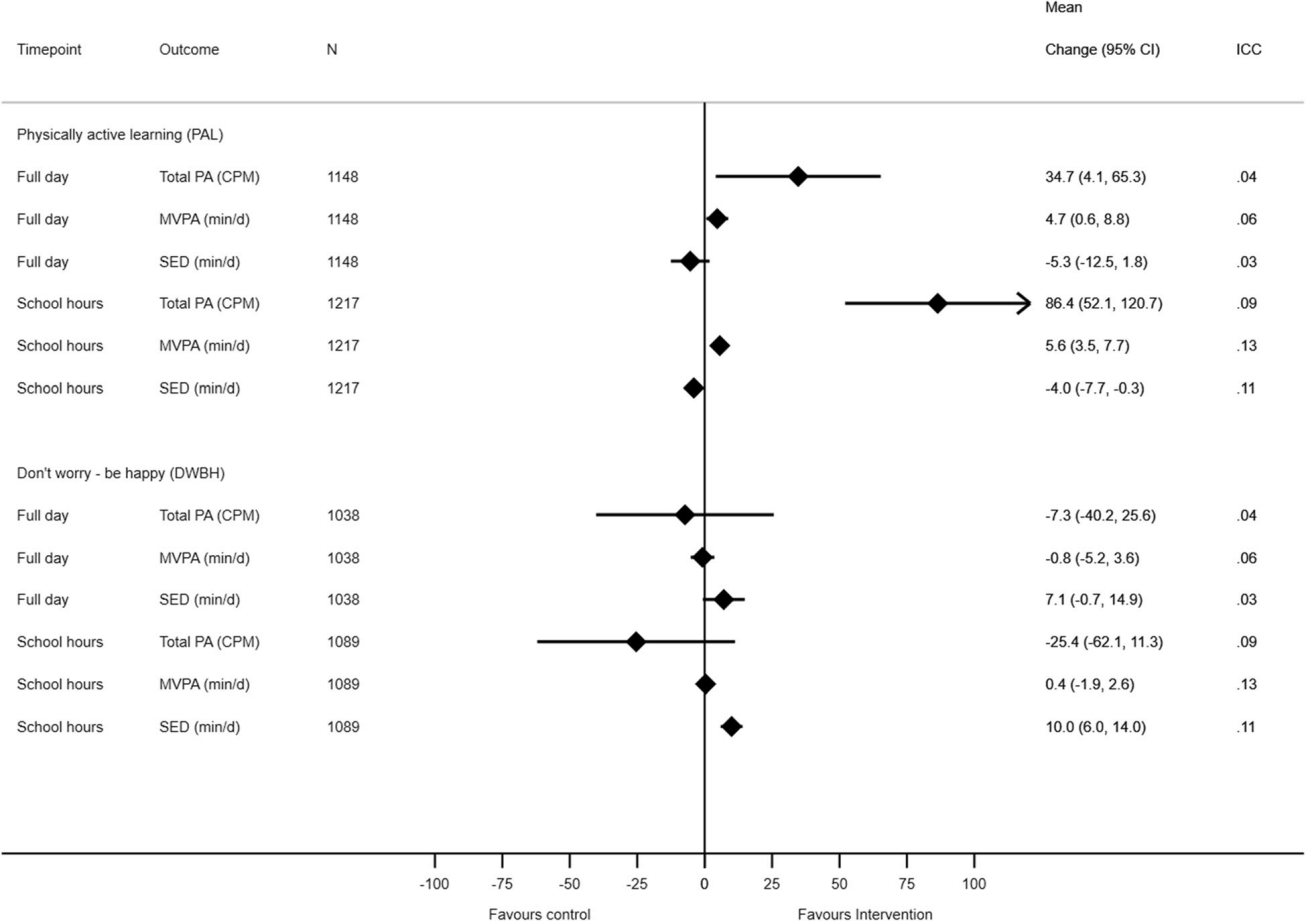
The intervention effect on physical activity variables during the *full day* and during school hours stratified by study group. MVPA = moderate- to vigorous-intensity physical activity, min = minutes, cpm = counts per minute, CI = confidence interval; ICC = intraclass correlation coefficient (for school). Each model contained fixed effects for intervention, time (baseline – follow-up) and intervention x time interaction, in addition to random effects for school, class and subject ID

We found significant between group differences in daily PA level and time spent in MVPA for adolescents in the PAL-intervention arm compared to adolescents in the control arm. The mean difference in change in PA level between participants in the PAL-intervention arm and the control arm was 34.7 cpm (95% CI: 4.1, 65.3) (Fig. 2). We observed a reduction in mean PA level during the intervention period among adolescents in both the intervention arms and control arm, however, the reduction was larger among controls (a reduction of 13 and 48 cpm, respectively). The mean difference in change in MVPA between participants in the PAL-intervention arm and the control arm was 4.7 min/day (95% CI: 0.6, 8.8). During the intervention period, time spent in MVPA decreased by 2.0 min/day for adolescents in the PAL-intervention, and by 6.7 min/day among adolescents in the control arm (Table 2 and Fig. 2). When the analyses were repeated stratified by gender, the intervention effect on both total PA level and time spent in MVPA was attenuated and no longer reached statistical significance (Table 4 and Additional file 3).

**Table 4:** The intervention effect on physical activity variables during the *full day* stratified by study arm and gender. The results are presented as mean differences in change (intervention arms vs controls) with 95% CI, *P*-values and ICC for school

	n	Mean difference in change (95% CI)^**a**^	*P*	ICC
**Girls**				
*PAL-intervention*				
Average PA (cpm)	624	27.6 (−6.8, 62.0)	0.116	0.05
MVPA (min/day)	624	4.7 (−0.5, 9.5)	0.052	0.06
Sedentary (min/day)	624	−5.6 (− 14.0, 2.7)	0.189	0.02
*DWBH-intervention*				
Average PA (cpm)	559	−11.8 (−49.4, 25.7)	0.537	0.05
MVPA (min/day)	559	1.7 (−6.9, 3.4)	0.510	0.06
Sedentary (min/day)	559	8.5 (−0.6, 17.7)	0.068	0.02
**Boys**				
*PAL-intervention*				
Average PA (cpm)	524	44.0 (−11.5, 100.8)	0.119	0.03
MVPA (min/day)	524	4.0 (−3.1, 11.2)	0.268	0.05
Sedentary (min/day)	524	−3.9 (−16.5, 8.7)	0.546	0.04
*DWBH-intervention*				
Average PA (cpm)	479	−3.3 (−62.7, 56.1)	0.913	0.03
MVPA (min/day)	479	0.1 (−7.5, 7.7)	0.981	0.05
Sedentary (min/day)	479	5.9 (−7.4, 19.3)	0.384	0.04

*PAL* Physically active learning; *DWBH* Don’t worry – Be happy, *PA* Physical activity, *MVPA* Moderate- to vigorous-intensity physical activity, *min* Minutes, *cpm* Counts per minute, *CI* Confidence interval, *ICC* Intraclass correlation coefficient

^a^Each model contained fixed effects for intervention, time (baseline – follow-up) and intervention x time interaction, in addition to random effects for school, class and subject ID

**Table 5:** The intervention effect on physical activity variables during *school hours* stratified by study group and gender. The results are presented as mean differences in change (intervention arms vs controls) with 95% CI, *P*-values and ICC for school

	n	Mean difference in change (95% CI)^**a**^	*P*	ICC
**Girls**				
*PAL-intervention*				
Average PA (cpm)	643	92.3 (52.2, 132.5)	**< 0.001**	0.10
MVPA (min/day)	643	6.3 (3.8, 8.8)	**< 0.001**	0.13
Sedentary (min/day)	643	−7.4 (−11.9, −2.8)	**0.001**	0.08
*DWBH-intervention*				
Average PA (cpm)	568	−9.4 (−53–0, 34.0)	0.670	0.10
MVPA (min/day)	568	1.0 (−1.6, 3.7)	0.462	0.13
Sedentary (min/day)	568	6.7 (1.8, 11.6)	**0.007**	0.08
**Boys**				
*PAL-intervention*				
Average PA (cpm)	574	76.6 (18.8, 134)	**0.009**	0.09
MVPA (min/day)	574	5.1 (1.5, 8.6)	**0.005**	0.13
Sedentary (min/day)	574	−0.5 (−6.6, 5.6)	0.878	0.12
*DWBH-intervention*				
Average PA (cpm)	521	−49.9 (− 110.5, 10.5)	0.106	0.09
MVPA (min/day)	521	−0.4 (−4.1, 3.3)	0.842	0.13
Sedentary (min/day)	521	13.2 (6.7, 19.8)	**< 0.001**	0.12

*PAL* Physically active learning; *DWBH* Don’t worry – Be happy; *PA* Physical activity; *MVPA* Moderate-to-vigorous physical activity, *min* Minutes, *cpm* Counts per minute, *CI* Confidence interval; *ICC* Intraclass correlation coefficient

^a^Each model contained fixed effects for intervention, time (baseline – follow-up) and intervention x time interaction, in addition to random effects for school, class and subject ID

We found no significant effect of the intervention on daily PA level, time spent in MVPA or sedentary time among adolescents in the DWBH-intervention arm compared with adolescents in the control arm (Fig. 2).

#### PA during school hours

During school hours, we found significant between group differences in PA level, MVPA and sedentary time among adolescents in the PAL-intervention arm compared to adolescents in the control arm (Fig. 2). The mean difference in change in PA level was 86.4 cpm (95% CI: 52.1, 120.7), 5.6 min/day of MVPA (95% CI: 3.5, 7.7) and − 4.0 min/day of sedentary time (95% CI: − 7.7, − 0.3). When the analyses were stratified by gender, we found similar significant effects in favor of the adolescents in the PAL-intervention arm, however, the favorable reduction in sedentary time was significant only among girls (Table 5 and Additional file 3).

When comparing adolescents in the DWBH-intervention arm with adolescents in the control arm, we found significant between group differences in sedentary time (Fig. 2). Both adolescents in the DWBH-intervention arm and in the control arm increased time spent sedentary during school hours throughout the intervention period, however, the increase was larger among adolescents in the DWBH-arm than among controls (mean difference 10.0 min/day, 95% CI: 6.0, 14.0). When we stratified the analyses by gender, we found similar significant effects on sedentary time in favor of the control arm in both girls and boys (Table 5 and Additional file 3).

### Secondary outcome – CRF and muscle strength

**Fig. 3:**
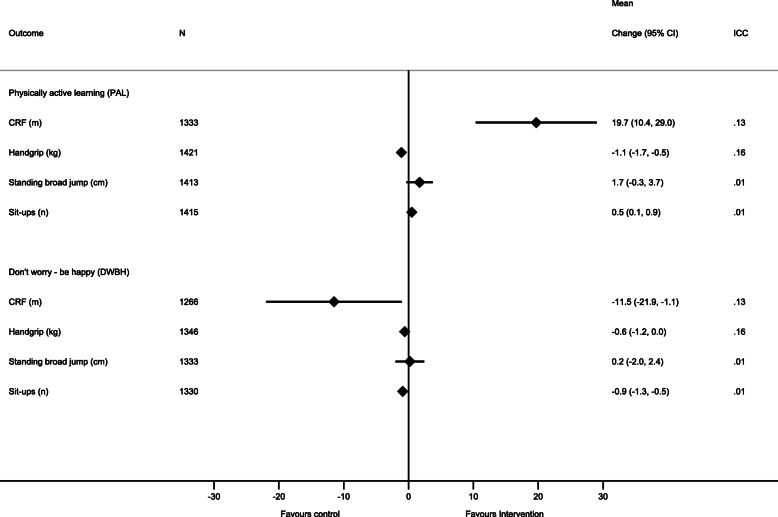
The intervention effect on cardiorespiratory fitness and muscle strength stratified by study group. Each model contained fixed effects for intervention, time (baseline – follow-up) and intervention x time interaction, in addition to random effects for school, class and subject ID

We found significant between group differences in CRF among adolescents in both intervention arms compared with adolescents in the control arm (Fig. 3). Both adolescents in the PAL-intervention arm and in the control arm increased distance run in the Andersen test during the intervention period. The mean difference in change was 19.8 m (95% CI: 10.4, 29.1) in favor of adolescents in the PAL-intervention arm compared to adolescents in the control arm. In the stratified analyses we observed significant effects on CRF among PAL-intervention boys but not among the girls when comparing with boys and girls in the control arm (Additional file 4).

During the intervention period, we found no change in the distance run in the Andersen test for the adolescents in the DWBH-intervention arm (Table 3). When comparing the change in distance run between the adolescents the DWBH intervention arm and the control arm, we found that the mean difference in change was 11.6 m (95% CI: − 22.0, − 1.1) in favor of the control arm (Fig. 3). The stratified analyses revealed that this unfavorable effect on CRF was observed among DWBH girls only (Table 6, Additional file 4).

**Table 6:** The intervention effect on cardiorespiratory fitness and muscle strength stratified by study arm and gender. The results are presented as mean differences in change (intervention arms vs controls) with 95% CI, *P*-values and ICC for school

	n	Mean difference in change (95% CI)^**a**^	*P*	ICC
**Girls**				
*PAL-intervention*				
Cardiorespiratory fitness (m)	656	3.2 (−10.3, 16.8)	0.643	0.16
Handgrip (kg)	698	**−1.8 (−2.5, − 1.1)**	**< 0.001**	0.24
Standing broad jump (cm)	693	**2.5 (0.1, 4.8)**	**0.036**	0.04
Sit-ups (n)	695	0.4 (−0.1, 1.0)	0.071	0.01
*DWBH-intervention*				
Cardiorespiratory fitness (m)	612	−24.6 (−39.8, −9.3)	**0.002**	0.16
Handgrip (kg)	648	**−0.8 (−1.5, − 0.1)**	**0.039**	**0.24**
Standing broad jump (cm)	644	−1.1 (−3.7, 1.4)	0.380	0.04
Sit-ups (n)	641	−0.2 (0.8, 0.3)	0.459	0.01
**Boys**				
*PAL-intervention*				
Cardiorespiratory fitness (m)	677	36.7 (24.0, 49.3)	**< 0.001**	0.09
Handgrip (kg)	723	−0.1 (−1.0, 0.7)	0.778	0.11
Standing broad jump (cm)	720	1.3 (−1.7, 4.4)	0.401	< 0.01
Sit-ups (n)	720	**0.6 (0.1, 1.2)**	**0.040**	0.07
*DWBH-intervention*				
Cardiorespiratory fitness (m)	654	1.4 (−12.6, 15.5)	0.842	0.09
Handgrip (kg)	697	−0.5 (−1.4, 0.3)	0.236	0.11
Standing broad jump (cm)	689	1.2 (−2.0, 4.4)	0.464	< 0.01
Sit-ups (n)	689	0.1 (−0.6, 0.6)	0.913	0.07

*PAL* Physically active learning; *DWBH* Don’t worry – Be happy; *ICC* Intraclass correlation coefficient,

^a^Each model contained fixed effects for intervention, time (baseline – follow-up) and intervention x time interaction, in addition to random effects for school, class and subject ID.,

We found significant between group differences in handgrip strength for adolescents in the PAL-intervention arm and adolescents in the DWBH-intervention arm compared to adolescents in the control arm (Fig. 3). All groups increased handgrip strength from baseline to follow-up; however, the increase was larger among adolescents in the control arm compared to adolescents in both intervention arms (mean difference PAL-intervention vs control: − 1.1 kg (95% CI: − 1.7, − 0.5) and mean difference DWBH-intervention vs control: − 0.6 kg (95% CI: − 1.3, 0.0) (Table 3).

We found significant between group differences in number of sit-ups performed for adolescents in the PAL-intervention arm compared to adolescents in the control arm (Fig. 3). The mean difference in change in sit-ups was 0.5 (95% CI: 0.1, 0.9) in favor of the students in the PAL-intervention arm. The intervention effect on the muscle strength tests stratified by gender is presented in Table 6 and Additional file 4.

## Discussion

This study assessed the effectiveness of two different school-based PA interventions on adolescents’ PA levels, cardiorespiratory fitness and muscle strength. After the intervention period, daily PA level and time spent in MVPA decreased in all groups, however the reduction was significantly smaller in the PAL-intervention group compared to the control group. The intervention was also effective in increasing CRF, where adolescents in the PAL-intervention arm increased significantly more than adolescents in the control arm. No intervention effects were observed on daily PA level, time spent in MVPA, or time spent sedentary among adolescents in the DWBH-intervention arm compared to adolescents in the control arm.

The PAL-intervention effect on the adolescents’ daily PA level agrees with some previous studies that have used accelerometers to assess PA, though there are conflicting results within the literature. Two multicomponent, school-based obesity prevention interventions that included adolescents from low-income areas found no effect on PA level after 20 weeks [17] and 12 months interventions [15]. Further, a 20-week after-school dance program for girls [16] and a six-week intervention study [31] did not increase 11–12-year-olds PA levels. A cohort of 12–13-year-old adolescents (*n* = 1440) in Ecuador completed a 28-month intervention on PA and physical fitness [18]. In a subsample (*n* = 226) it was observed that whilst more than 90% of the cohort achieved more than 60 min of MVPA per day at baseline, the proportion decreased during the intervention period. Yet, the decrease was significantly lower in the intervention group compared to the control arm (6 vs 18 percentage points) [18]. Similar results were also reported among Australian 14-year-olds (*n* = 1150) after the completion of a 24-month school-based PA-intervention [19]. The intervention was effective in increasing daily MVPA in the intervention group compared with a decrease in the control group. Even though the results from the two latter studies have comparable findings as ours, it should be mentioned that the adolescents in these studies came from a middle-income country or disadvantaged communities and are therefore not directly comparable to the adolescents in our study. Further, both studies which reported positive results were long lasting interventions with durations of almost 2 years, including comprehensive approaches to promote an active lifestyle. Our intervention lasted 9 months and targeted the adolescents’ PA levels during school hours only. We therefore speculate that had we implemented the intervention over two school years and also targeted leisure time activities in the home and neighborhood environment the intervention effect could have been stronger [32]. When stratifying the analyses by gender, the positive intervention effect on both total PA level and time spent in MVPA during the full day remained positive but were no longer statistically significant as the study was not powered for stratified analyses. The mean PA level in our cohort is quite high compared to similar age groups in other European countries [2]. However, relatively large reductions in mean PA level are observed when going from childhood through adolescence [4], hence it is important to implement PA interventions which aim to curb this decline. In the PAL-intervention, the decreases in both mean PA and time spent in MVPA were significantly smaller. On average, MVPA in the PAL-intervention group decreased by 2 min/day, whilst in the control arm MVPA decreased by 6 min/day. Though this between-group difference seems small, it translates to 28 min/week more MVPA in the PAL-intervention group. Hence, the PAL-intervention can be considered as one approach to curb the decline in MVPA.

Positive and stronger intervention effects on PA-level were observed during school hours. Adolescents in the PAL-intervention arm increased their mean PA level while time spent in MVPA was stable. A decrease in both mean PA and time spent in MVPA was observed among adolescents in the DWBH-intervention arm and the control arm. The latter finding is not a surprise, as PA levels are expected to decrease through adolescence [1, 3]. The fact that the adolescents in the PAL-intervention arm maintained and even increased their PA-level during school hours must therefore be considered a strong and positive result of the intervention. Further, this indicates that the positive intervention effect observed on PA levels depend on how the additional PA was implemented. In the PAL-intervention, we encouraged the teachers to engage the students in activities of at least moderate intensity, however, this was not the focus in the DWBH-intervention. In the DWBH-intervention we promoted friendships through PA and PE, and we considered the social relationships more important than the intensity of the activity. However, as the students were able to choose their activities based on their interest and preference, our expectation was that this would stimulate more PA. Some of the adolescents in the DWBH-intervention arm performed activities like football, walking, or bicycling that includes movements of some intensity, but other groups chose activities that were less physically demanding (for instance yoga or building tree houses in the woods). Further, although accelerometers have been shown to provide valid and reliable estimates of PA level in children and adolescents, they have some limitations including the ability to detect certain activities and upper body movements [33]. Consequently, some activities in the DWBH-intervention arm, such as cycling or resistance training, may have been underestimated. In the DWBH-intervention the students themselves led and organized the activities, whilst the PAL-intervention was led and taught by teachers. We speculate that students tend to engage in lower intensity activities if they themselves can choose the intensity, although the intensity will also be highly dependent on the chosen activity and the individual. It should also be mentioned that at baseline adolescents in the PAL-intervention arm were less physically active than adolescents in the control arm, thus, it could be that the intervention potential was higher in this group. However, the linear mixed models used to analyze the intervention effects incorporated baseline values, therefore, baseline differences cannot explain the positive results. Lastly, both adolescents in the DWBH-intervention arm and the control arm increased time spent sedentary during the intervention period, but the increase was significantly higher in the DWBH-intervention arm. We attribute this counterintuitive finding to the fact that our intervention promoted PA and did not target sedentary time specifically.

The positive effect on CRF observed among adolescents in the PAL-intervention arm is in keeping with some results from previous school-based interventions, even though most of these were carried out among elementary school children [34]. The PAL-intervention resulted in an improvement of running distance of 3.5% compared to baseline values, whereas the DWBH-intervention resulted in no change in running distance compared to baseline values. Even though the observed PAL-intervention effect on CRF is modest, it is relevant as high CRF in adolescence is of importance for future health [35]. The stratified analyses revealed greater improvements in running distance among PAL-intervention boys than among PAL-intervention girls compared to controls. Sex differences in CRF among adolescents are attributed to a number of factors like lean body mass, hemoglobin concentration and testosterone among others [36]. During puberty, boys experience increases in testosterone production and muscle mass that is higher than in girls and these factors are beneficial for CRF. Girls on the other hand experience increases in fat mass which often results in a stagnation in performance-related tests, such as the Andersen test, where body weight and body composition are of importance.

All groups increased their hand grip strength during the intervention period; however, the increase was greater among the adolescents in the control group. Furthermore, a positive intervention effect was observed on sit-ups among adolescents in the PAL-intervention compared to the control group. Even though both mentioned intervention effects were significant, they were small and of little relevance. Mixed intervention effects on muscle strength were also observed in the study including adolescents in Ecuador [18]. The intervention increased vertical jump, however, the adolescents in the control group needed less attempts to keep their balance for 1 min in Flamingo balance test compared to intervention group. The finding of small intervention effects on muscle strength is not a surprise. The PAL-intervention did not target strength training in particular. In the DWBH-intervention, some of the groups performed activities that could theoretically improve muscle strength (like groups doing resistance training or yoga), however, no effect was observed on group level.

This study has several strengths, including use of a cluster RCT design, use of accelerometers to assess PA, the implementation of two separate PA interventions aimed at increasing PA levels and a large cohort. Approximately 76% of the study population consented to participate which reduced the risk of sampling bias. Also, randomization occurred on the school level to reduce risk of contamination, and the cluster RCT design was accounted for in the analyses. The two intervention arms were pilot tested during the school year 2016–17 in seven lower secondary schools (including approximately 700 students). Based on the pilot study, some adjustments were made to both intervention arms. These adjustments tailored the intervention to better reach the target group. Further, both intervention arms were designed to require only modest changes in already existing school structures, and none of the intervention components required expensive equipment. The intervention was delivered by teachers or the students themselves, which is a sustainable approach. Also, the intervention delivery was approximately 80% on average during the intervention period and indicates that the intervention models work well in real life contexts. The simplicity of both interventions would likely make it possible to adopt and implement in other lower secondary schools in Norway.

A limitation of the study is the loss of students with PA-assessments at follow-up, with less than half of students that initially consented providing accelerometer data at follow-up, a finding consistent with previous studies [15, 19]. The loss to follow-up was greater for accelerometer data than for the physical fitness data. As the adolescents were asked to wear the accelerometer for 7 days, they might have experienced this a burden and therefore chose not to wear it. Compliance may be improved by the provision of compensation strategies such as monetary incentives for wearing the accelerometer or for correct wear [37]. In the analyses, we used statistical methods (mixed models) that considered the loss to follow-up, consequently, all students who had valid accelerometer data at one time point were included in the analyses to reduce the loss of statistical power. Also, both intervention arms were designed to engage the adolescents in an additional 120 min of PA per week. However, for some of the components this included time to organize activities and for the adolescents to change clothes and shower, consequently, the intervention dose is probably somewhat lower than 120 min. Lastly, during school days we defined a valid day of accelerometer wear time as at least 40% of accelerometer data during school hours. This might seem low; however, few school-based intervention studies have used individual timetables for each school to create classifications and the basis for comparison is minimal. Therefore, we used approximately the same wear time criteria as during a full day, where a valid day was defined as at least 8 h, which corresponds to 44% of the awake time (6 AM to midnight).

## Conclusions

During the nine-month intervention period, we observed a smaller decrease in daily PA level, time spent in MVPA, as well as improvement in CRF among adolescents in the PAL-intervention arm compared to adolescents in the control arm. During school hours, we observed increased PA levels and reduced sedentary time among adolescents in the PAL-intervention arm compared to adolescents in the control arm. We found no intervention effect on daily PA levels among adolescents in the DWBH-intervention arm compared to controls. We did, however, observe unfavorable intervention effects on time spent sedentary during school hours as well as CRF in the DWBH-intervention arm compared to controls. The PAL-intervention model was teacher-led focusing on the dose and intensity of PA, whereas the DWBH-intervention arm was a social intervention, being student-led and emphasizing the social aspects of PA. Our results indicate that a teacher-led intervention including three unique intervention components, where activities of at least moderate intensity was encouraged, is effective in curbing a decline in PA and improving CRF compared to controls.

## Supplementary Information

**Additional file 1:**. CONSORT 2010 checklist of information to include when reporting a randomised trial*.

**Additional file 2:**. The TIDieR (Template for Intervention Description and Replication) Checklist*.

**Additional file 3: Table 2a**. Mean (95% confidence interval) physical activity levels among girls stratified by study arm at baseline and follow-up. **Table 2b.** Mean (95% confidence interval) physical activity levels among boys stratified by study arm at baseline and follow-up.

**Additional file 4: Table 3**. Mean (95% confidence interval) for cardiorespiratory fitness and muscle strength among participants stratified by study arm and gender at baseline and follow-up.

## Data Availability

The datasets generated and/or analyzed during the current study are not publicly available as publications are planned but are available from the corresponding author on reasonable request.

## Abbreviations

DWBH: Don’t worry – be happy
MVPA: Moderate-to-vigorous intensity physical activity
PA: Physical activity
PAL: Physically active learning
PE: Physical education
RCT: Randomized controlled trial
ScIM: School in motion
