# Supplementary material for: The effect of a school-based intervention on physical activity, cardiorespiratory fitness and muscle strength: the School in Motion cluster randomized trial
Source: Int J Behav Nutr Phys Act. 2020 Nov 26;17:154. doi: 10.1186/s12966-020-01060-0 (PMC7690135; doi:10.1186/s12966-020-01060-0)
Supplement: Supplementary file 3 — Additional file 3: Table 2a. Mean (95% confidence interval) physical activity levels among girls stratified by study arm at baseline and follow-up. Table 2b. Mean (95% confidence interval) physical activity levels among boys stratified by study arm at baseline and follow-up. [file 12966_2020_1060_MOESM3_ESM.docx]

**Additional table 2a.** Mean (95% confidence interval) physical activity levels among girls stratified by study arm at baseline and follow-up.

|  | **PAL-intervention** | | | |  | **DWBH-intervention** | |  | **Control** | |
| --- | --- | --- | --- | --- | --- | --- | --- | --- | --- | --- |
|  |  | **Baseline** | **Follow-up** | |  | **Baseline** | **Follow-up** |  | **Baseline** | **Follow-up** |
| **Physical activity**  **levels full day** | | | | | | | | | | |
| N |  | 273 | 193 | |  | 216 | 133 |  | 308 | 175 |
| Wear time (min/day) |  | 781 (769, 793) | 770 (756, 783) | |  | 782 (769, 796) | 779 (763, 795) |  | 777 (766, 788) | 748 (735, 762) |
| Average PA (cpm) |  | 475 (441, 509) | 454 (418, 490) | |  | 510 (473, 547) | 450 (410, 490) |  | 502 (473, 547) | 454 (417, 491) |
| MVPA (min/day) |  | 64 (59, 69) | 62 (57, 68) | |  | 69 (63, 74) | 61 (55, 66) |  | 68 (63, 72) | 61 (56, 67) |
| Sedentary time (min/day) |  | 556 (549, 562) | 566 (559, 573) | |  | 546 (538, 553) | 570 (562, 579) |  | 545 (538, 551) | 560 (553, 568) |
|  |  |  |  |  |  |  |  |  |  |  |
| **Physical activity**  **school hours** | | | | | | | | | | |
| N |  | 287 | 214 | |  | 222 | 152 |  | 309 | 203 |
| Wear time (min/day) |  | 321 (311, 330) | 316 (306, 326) | |  | 322 (311, 332) | 327 (316, 338) |  | 314 (304, 324) | 295 (284, 305) |
| Average PA (cpm) |  | 393 (354, 439) | 412 (368, 456) | |  | 462 (416, 508) | 375 (327, 423) |  | 459 (415, 502) | 381 (336, 427) |
| MVPA (min/day) |  | 22 (19, 25) | 23 (20, 26) | |  | 26 (23, 29) | 22 (18, 25) |  | 26 (23, 29) | 21 (18, 24) |
| Sedentary time (min/day) |  | 235 (231, 240) | 236 (231, 241) | |  | 225 (220, 230) | 240 (235, 246) |  | 228 (223, 233) | 236 (231, 241) |
| PAL = Physically active learning; DWBH = Don’t Worry – Be Happy, cpm = counts per minute; MVPA = moderate- to vigorous-intensity physical activity. All analyses are adjusted for wear time (except cpm), school cluster, class cluster and subject ID as random effect. | | | | | | | | | | |

|  | **PAL-intervention** | | | |  | **DWBH-intervention** | |  | **Control** | |
| --- | --- | --- | --- | --- | --- | --- | --- | --- | --- | --- |
|  |  | **Baseline** | **Follow-up** | |  | **Baseline** | **Follow-up** |  | **Baseline** | **Follow-up** |
| **Physical activity**  **levels full day** | | | | | | | | | | |
| N |  | 227 | 124 | |  | 190 | 96 |  | 258 | 113 |
| Wear time (min/day) |  | 769 (754, 785) | 755 (737, 774) | |  | 764 (747, 780) | 752 (732, 773) |  | 763 (748, 778) | 735 (715, 754) |
| Average PA (cpm) |  | 550 (512, 589) | 550 (504, 595) | |  | 561 (519, 603) | 513 (462, 563) |  | 584 (545, 622) | 538 (491, 586) |
| MVPA (min/day) |  | 71 (65, 76) | 68 (62, 75) | |  | 72 (66, 78) | 66 (59, 73) |  | 76 (71, 82) | 69 (63, 76) |
| Sedentary time (min/day) |  | 522 (512, 533) | 539 (527, 551) | |  | 519 (508, 531) | 546 (532, 559) |  | 513 (502, 523) | 533 (521, 545) |
|  |  |  |  |  |  |  |  |  |  |  |
| **Physical activity**  **school hours** | | | | | | | | | | |
| N |  | 249 | 161 | |  | 205 | 132 |  | 274 | 150 |
| Wear time (min/day) |  | 324 (314, 335) | 320 (309, 331) | |  | 320 (309, 331) | 218 (206, 239) |  | 314 (303, 324) | 288 (277, 299) |
| Average PA (cpm) |  | 493 (437, 548) | 517 (458, 575) | |  | 554 (494, 613) | 451 (387, 515) |  | 557 (501, 612) | 504 (443, 565) |
| MVPA (min/day) |  | 27 (23, 31) | 28 (24, 32) | |  | 31 (27, 35) | 26 (22, 30) |  | 32 (28, 36) | 28 (24, 32) |
| Sedentary time (min/day) |  | 217 (211, 224) | 223 (216, 230) | |  | 208 (201, 215) | 228 (220, 235) |  | 211 (204, 217) | 217 (209, 224) |
| PAL = Physically active learning; DWBH = Don’t Worry – Be Happy, cpm = counts per minute; MVPA = moderate- to vigorous-intensity physical activity. All analyses are adjusted for wear time (except cpm), school cluster, class cluster and subject ID as random effect. | | | | | | | | | | |

**Additional table 2b.** Mean (95% confidence interval) physical activity levels among boys stratified by study arm at baseline and follow-up.
