# Supplementary material for: The effect of a school-based intervention on physical activity, cardiorespiratory fitness and muscle strength: the School in Motion cluster randomized trial
Source: Int J Behav Nutr Phys Act. 2020 Nov 26;17:154. doi: 10.1186/s12966-020-01060-0 (PMC7690135; doi:10.1186/s12966-020-01060-0)
Supplement: Supplementary file 4 — Additional file 4: Table 3. Mean (95% confidence interval) for cardiorespiratory fitness and muscle strength among participants stratified by study arm and gender at baseline and follow-up. [file 12966_2020_1060_MOESM4_ESM.docx]

|  | **PAL-intervention** | | |  | **DWBH-intervention** | |  | **Control** | |
| --- | --- | --- | --- | --- | --- | --- | --- | --- | --- |
|  |  | **Baseline** | **Follow-up** |  | **Baseline** | **Follow-up** |  | **Baseline** | **Follow-up** |
| **Girls** |  | | | | | | | | |
|  |  |  |  |  |  |  |  |  |  |
| Cardiorespiratory fitness (m) |  | 867 (841, 894) | 887 (860, 914) |  | 887 (859, 914) | 878 (851, 906) |  | 908 (881, 935) | 925 (897, 952) |
| Handgrip (kg) |  | 29 (27, 31) | 29 (27, 30) |  | 28 (26, 30) | 28 (26, 30) |  | 27 (25, 29) | 29 (27, 31) |
| Standing broad jump (cm) |  | 160 (155, 164) | 163 (159, 167) |  | 163 (159, 167) | 163 (158, 167) |  | 166 (161, 170) | 166 (162, 171) |
| Sit, ups (n) |  | 17 (16, 18) | 18 (17, 19) |  | 17 (16, 18) | 17 (16, 18) |  | 18 (17, 19) | 19 (18, 20) |
|  |  |  |  |  |  |  |  |  |  |
| **Boys** | | | | | | | | | |
| Cardiorespiratory fitness (m) |  | 918 (892, 945) | 962 (936, 989) |  | 933 (906, 961) | 942 (914, 970) |  | 949 (923, 975) | 956 (930, 982) |
| Handgrip (kg) |  | 31 (29, 33) | 36 (33, 38) |  | 32 (29, 34) | 36 (34, 38) |  | 31 (29, 33) | 36 (33, 38) |
| Standing broad jump (cm) |  | 177 (173, 180) | 180 (186, 193) |  | 179 (175, 183) | 191 (187, 195) |  | 179 (176, 182) | 190 (187, 193) |
| Sit, ups (n) |  | 19 (18, 20) | 20 (19, 21) |  | 19 (18, 20) | 20 (19, 21) |  | 20 (19, 21) | 21 (20, 22) |
| PAL = Physically active learning; DWBH = Don’t Worry – Be Happy. All analyses are adjusted for school cluster, class cluster and subject ID as random effect. | | | | | | | | | |

**Additional table 3.** Mean (95% confidence interval) for cardiorespiratory fitness and muscle strength among participants stratified by study arm and gender at baseline and follow-up.
